# Altered trends of local brain function in classical trigeminal neuralgia patients after a single trigger pain

**DOI:** 10.1186/s12880-024-01239-y

**Published:** 2024-03-18

**Authors:** Juncheng Yan, Luoyu Wang, Lei Pan, Haiqi Ye, Xiaofen Zhu, Qi Feng, Haibin Wang, Zhongxiang Ding, Xiuhong Ge

**Affiliations:** 1 Department of Rehabilitation, Hangzhou First People’s Hospital, 310000 Hangzhou, China; 2 Department of Radiology, Hangzhou First People’s Hospital, 310000 Hangzhou, China; 3 Department of Radiology, Key Laboratory of Clinical Cancer Pharmacology and Toxicology Research of Zhejiang Province, Cancer Center, Hangzhou First People’s Hospital, 310006 Hangzhou, China

**Keywords:** *Classical trigeminal neuralgia*, *Regional homogeneity*, *Dynamic*, *Time-frequency*, *Pathogenesis*, *Resting-state functional magnetic resonance imaging*

## Abstract

**Objective:**

To investigate the altered trends of regional homogeneity (ReHo) based on time and frequency, and clarify the time-frequency characteristics of ReHo in 48 classical trigeminal neuralgia (CTN) patients after a single pain stimulate.

**Methods:**

All patients underwent three times resting-state functional MRI (before stimulation (baseline), after stimulation within 5 s (triggering-5 s), and in the 30th min of stimulation (triggering-30 min)). The spontaneous brain activity was investigated by static ReHo (sReHo) in five different frequency bands and dynamic ReHo (dReHo) methods.

**Results:**

In the five frequency bands, the number of brain regions which the sReHo value changed in classical frequency band were most, followed by slow 4 frequency band. The left superior occipital gyrus was only found in slow 2 frequency band and the left superior parietal gyrus was only found in slow 3 frequency band. The dReHo values were changed in midbrain, left thalamus, right putamen, and anterior cingulate cortex, which were all different from the brain regions that the sReHo value altered. There were four altered trends of the sReHo and dReHo, which dominated by decreased at triggering-5 s and increased at triggering-30 min.

**Conclusions:**

The duration of brain function changed was more than 30 min after a single pain stimulate, although the pain of CTN was transient. The localized functional homogeneity has time-frequency characteristic in CTN patients after a single pain stimulate, and the changed brain regions of the sReHo in five frequency bands and dReHo complemented to each other. Which provided a certain theoretical basis for exploring the pathophysiology of CTN.

**Supplementary Information:**

The online version contains supplementary material available at 10.1186/s12880-024-01239-y.

## Introduction

Trigeminal neuralgia (TN) was a chronic neurogenic pain which was often triggered by harmless actions in daily life and the pain lasted for a few seconds to several minutes [1–4], there was no pain attack during the inter-phase [5]. The prevalence of TN in the general population ranged from 0.016–0.3%[6,[7]], the incidence increased with age and was common in women over 50 years old [8]. TN could seriously affect the patients’ quality of life, included anxiety, depression, and even suicide [3, 9]. According to the third edition of the International Classification of Headache Disorders (ICHD-3), the TN was divided into classical TN (CTN), secondary TN (STN), and idiopathic TN (ITN) [10], the STN could secondary to tumor [10, 11].

Neurovascular compression (NVC) on the trigeminal nerve root [4, 7] was hypothesized to be the etiology of CTN, which may relate to ectopic impulses caused by demyelination and regeneration of trigeminal nerve after compression [12]. But some healthy controls (HCs) and the unaffected side of CTN also have NVC [13], so the NVC may be only one of the important factors of CTN. And, studies found that the brain function of CTN patients were changed [2, 14–17], which may be one of the pathogenic factors of CTN. Functional magnetic resonance imaging (fMRI) has been applied to investigate the brain function and structure of CTN [15, 18–26].

Neuroimaging had the advantages of noninvasive and convenient, and the resting-state function magnetic resonance imaging (rs-fMRI) captured the neural activity in the brain of participants at rest, more closely mirroring the physiological state [27], which could reflect the physiological state of body, the progression of disease, and understand disease status [28]. The static regional homogeneity (sReHo) reflected the temporal synchronization of brain activity in resting state [29, 30], and was a highly reliable feature of human brain connectivity [28, 31]. Although the rs-fMRI was acquired in the resting state, spontaneous brain activity was not completely stationary and changed over time [32]. Dynamical regional homogeneity (dReHo) was an rs-fMRI analysis method and captured the temporal variability of sReHo, the brain regions which with large fluctuations in dReHo were considered to be the functional center of brain [33]. Ge et al. [34] found that the sReHo and dReHo of CTN patients were all changed in different brain regions compared with HCs, and the altered brain regions of the two indicators were complementary, but they did not study the local brain function of pain process or before and after the pain.

According to the previous studies, the frequency could divide into four sub-bands: slow 5 frequency band (0.01~0.027 Hz) reflects the activity of cortical neurons, slow 4 frequency band (0.027~0.073 Hz) reflects the activity of basal ganglia, slow 3 frequency band (0.073 ~ 0.198 Hz) related to physiological noise, and slow 2 frequency band (0.198 ~ 0.25 Hz) related to white matter signals, the slow 4 and slow 5 frequency bands were included in the classical frequency band (0.01~0.08 Hz) [35–37], the frequency components of rs-fMRI signal could provide a new set of functional brain markers [34, 36–38]. In our previous study, we compared the brain function of CTN and HCs in three frequency bands, and found that the changed brain regions had frequency characteristic. Although CTN had frequency feature compared with HCs, but whether this feature existed in the pain process was unknown, which may provide important information about the pathophysiological process of CTN.

In order to clarify the altered trends and time-frequency characteristics of regional homogeneity, we measured sReHo values in five frequency bands (classical, slow 5, slow 4, slow 3, and slow 2 equivalent to frequency characteristic) and dReHo values (equivalent to time characteristic) at three time points before and after a single pain stimulate in CTN patients. Our hypotheses were that: (1) in different frequency band, the brain regions which sReHo changed were overlapped and complementary; (2) the changed trend of the same brain region was consistent in different frequency band; (3) the changed brain regions of dReHo and sReHo were different, which could provide beneficial supplement to sReHo.

## Materials and methods

This study was approved by the local ethics committee of Hangzhou First People’s Hospital (IRB# NO.202,107,002) and was carried out following the Declaration of Helsinki. All the participants provided written informed consent.

### Participants

A total of 85 CTN patients were recruited from our Hospital. The inclusion criteria for patients with CTN were as follows: (1) the CTN patients were diagnosed according to the ICHD-3^10^; (2) unilateral pain; (3) paroxysmal facial pain precipitated by trigger factors; (4) conventional magnetic resonance imaging examination revealing no evidence of abnormal brain signals; (5) no additional neurological or sensory deficits in all patients; (6) no previous surgical or other invasive procedures for CTN; (7) no contraindications to MRI scanning; (8) patients underwent microvascular decompression and confirmed that the NVC was existed; and (9) right-handness [39].

The exclusion criteria were as follows: (1) patients with CTN who had undergone surgical treatment before; (2) headaches and other paroxysmal or chronic pain conditions; (3) a family history of headache or other pain in first-degree relatives; (4) other somatic or psychiatric conditions; (5) contraindications to MRI; and (6) left handedness [39].

### Experimental design

Patients were asked to stop their analgesic medications 12 h before scheduled scanning sessions. Before the MRI scan, a medical history was taken to confirm the trigger zone. Stimulated the trigger zone within 5 s before the second rs-fMRI scan, the trigger zone was stimulated gently by the doctor using the long cotton swab [23]. The foam was used for head fixation to ensure that the patient remained head-still during the scan. All participants underwent 3D-T1WI and three times rs-fMRI. The three times rs-fMRI was performed before stimulating the trigger zone (baseline), within 5 s after stimulating the trigger zone (triggering-5s), and in the 30th minute after stimulating the trigger zone (triggering-30 min). After MRI scanning, the patients were asked whether the stimulation caused pain and if experienced additional pain during the scan [39].

### Pain evaluation

The pain degree of CTN patients was assessed by the visual analogue scale (VAS). The researcher guided the patient in rating their pain on a scale of 0–10. A higher score indicated greater pain intensity. A rating of “0” represented no pain, and a rating of “10” meant intolerable pain [39].

### MRI parameters

All participants underwent MRI using a 3.0T MRI scanner (Siemens, MAGNETOM Verio, Germany) with an eight-channel phased-array head coil. Participants were instructed to close their eyes, stay awake, and breathe quietly during the scanning process [39]. The imaging parameters for structural images were as follows: repetition time (TR) = 1900 ms, echo time (TE) = 2.52 ms, thickness = 1 mm, field of view (FOV) = 256 × 256 mm [2], voxel size = 1 × 1 × 1 mm [3], and turning angle = 9 degree. For functional images, the parameters were: TR = 2000 ms, TE = 30 ms, thickness = 3.2 mm, voxel size = 3.44 × 3.44 × 3.20 mm [3], turning angle = 90 degree, FOV = 220 × 220 mm [2], and scan time = 8 min.

### Image preprocessing

Rs-fMRI data preprocessing was performed using Data Processing and Analysis of Brain Imaging (DPABI6.1) and Statistical Parametric Mapping 12 (SPM12) toolbox based in MATLAB (MathWorks, MA, USA). Preprocessing steps included discarded the first 10 volumes to ensure MRI signal reached a steady state, slice-timing and head motion correction, normalization to the Montreal Neurological Institute (MNI) space with 3 × 3 × 3 mm [3] voxel size, detrended the BOLD signal, and noise removal through regression of Friston-24 head motion parameters, cerebrospinal fluid signals, and white matter signals. Four patients were excluded due to large head motion (more than 3-mm maximum displacement, 3° rotation, or the framewise displacement exceeding 0.5 throughout the course of scanning), and the remaining 48 CTN patients were selected for further analysis (**Figure S3**).

### sReHo calculation

After preprocessing, band-pass filtering was applied in five frequency bands (classical frequency band, 0.01~0.08 Hz; slow 2, 0.198~0.025 Hz; slow 3, 0.073~0.198 Hz; slow 4, 0.027~0.073 Hz; and slow 5, 0.01~0.027 Hz). A whole-brain ReHo analysis was conducted for each participant in different frequency bands by calculating Kendall’s coefficient of concordance (KCC) between a given voxel’s time series and its 26 closest voxels. Spatial smoothing was applied using a Gaussian kernel with 6 mm full width at half maximum.

### dReHo calculation

The sliding window method was used for dReHo calculation to detect temporal fluctuations in brain regions. The brain regions that exhibit substantial fluctuations typically in dReHo were considered as the functional centers of the brain [23]. Hence, the choice of window length was an critical parameter in calculating resting-state dynamics. Striking the right balance in window length was crucial to capture meaningful and reliable changes in brain activity over time [29]. Window length was an important parameter in the calculation of dynamic indicators, shorter window length may increase the risk of introducing spurious fluctuations in the observed dReHo, and longer window length may hinder the description of the time variability dynamics of ReHo [29]. The minimum window length should be larger than 1/f min, where f min was defined as the minimum frequency of the time series [40], in this way, spurious fluctuations could be excluded. So, a sliding window length of 50TR (100 s) and a moving step size of 2TR (4 s) were used. Multiple window-based ReHo maps were generated for each participant, and the standard deviation (SD) was calculated to measure the dynamics of ReHo [40]. The step size of 1 TRs (2 s) was further applied to validate the results of dReHo with the different step sizes (Table **S1**, **Figure **S1  and S2).

### Statistical analysis

Data Processing & Analysis of Brain Imaging (DPABI) software was used to compare sReHo and dReHo values, and measure the values in CTN patients at three times point. Repeated-measures analysis of variance (ANOVA) was conducted to examine the differences between groups. Gauss random field theory (GRF) was applied for multiple comparison correction, with significance thresholds set at *P* < 0.001 (cluster level) and *P* < 0.001 (voxel level). Spearman correlations were performed on pain characteristics (disease duration, VAS, pain frequency) and the sReHo and dReHo values.

## Results

### Demographic information and clinical characteristics

A total of 85 CTN patients participated in the scanning, and finally 48 patients were included in this study. The details of the 48 CTN patients were consistent with our previous study [39]. The inclusion and exclusion procedures were shown in **Figure S3**. The course of the disease, distribution of pain, duration of each pain episode, and pain score were shown in Table S2.

### sReHo changed in the five frequency bands after triggering pain in CTN patients

**Fig. 1:**
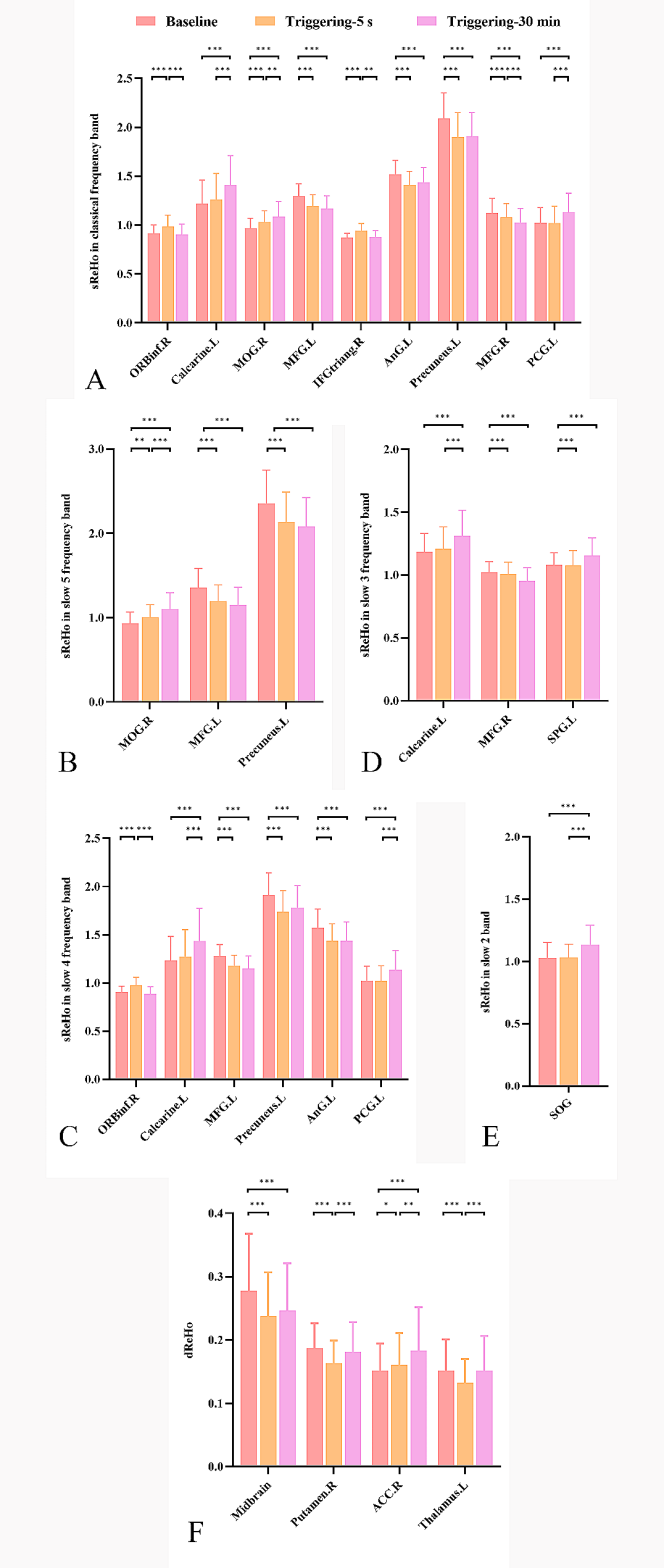
The sReHo changed in different frequency bands and dReHo changed in classical frequency band after a single pain stimulate in CTN patients. **A** classical frequency band, **B** slow 5 frequency band, **C** slow 4 frequency band, **D** slow 3 frequency band, **E** slow 2 frequency band, **F** dReHo. *sReHo, Static regional homogeneity; dReHo, Dynamic regional homogeneity; CTN, classical trigeminal neuralgia*

**Fig. 2:**
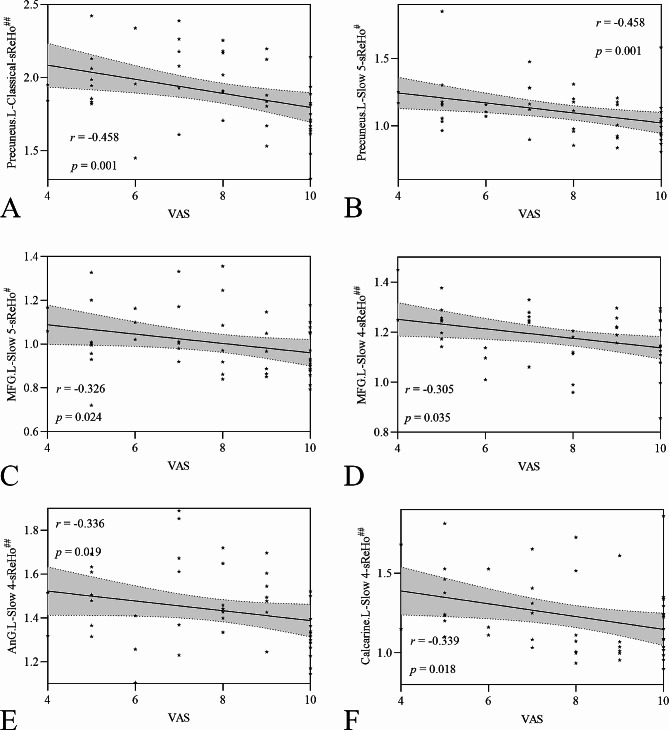
Post hoc comparisons of analysis of variance. The connection between two bars represents significant between-time differences of sReHo in five frequency bands and dReHo (^*^represents significant level *P* < 0.05, ^**^denotes significant level *P* < 0.01, and ^***^ indicates significant level *P* < 0.001, Bonferroni correction). **A** classical frequency band, **B** slow 5 frequency band, **C** slow 4 frequency band, **D** slow 3 frequency band, **E** slow 2 frequency band, **F** dReHo. *sReHo, static regional homogeneity; dReHo, Dynamic regional homogeneity; baseline, the rs-fMRI was performed before stimulating the trigger zone; triggering-5 s, the rs-fMRI was performed within 5 s after stimulating the trigger zone; triggering-30 min, the rs-fMRI was performed in the 30th minute after stimulating the trigger zone; ORBinf.R, right inferior frontal gyrus, orbital part; Calcarine.L, left calcarine; MOG.R, right iddle occipital gyrus; MFG.L, left middle frontal gyrus; IFGtriang.R, right inferior frontal gyrus, triangular part; AnG.L, left angular gyrus; Precuneus.L, left precuneus; MFG.R, right middle frontal gyrus; PCG.L, left postcentral gyrus; SPG.L, left superior parietal gyrus; SOG.L, left superior occipital gyrus; Putamen.R, right putamen; ACC.R, right anterior cingulate cortex; Thalamus.L, left thalamus*

The number of the altered brain regions of classical frequency band was most, followed by the slow 4, slow 5, slow 3, and slow 2 frequency band. There were nine altered brain regions in classical frequency band, which had four changed trend, dominated by decreased at triggering-5 s and increased at triggering-30 min (left angular gyrus, precuneus, and postcentral gyrus), the left calcarine and right middle occipital gyrus was gradually increased, while sReHo in bilateral middle frontal gyrus were gradually decreased at triggering-5 s and triggering-30 min, and the right inferior frontal gyrus, orbital part, and inferior frontal gyrus, triangular part were increased at triggering-5 s and decreased at triggering-30 min (Table 1 and S3, Figs. 1 and 2).

**Table 1:** The static regional homogeneity difference in five frequency bands in CTN patients after triggering pain

Bands	Brain region	Side	Peak MNI coordinates			Cluster size (voxels)	Peak intensity	F value	P value	Post hoc P value		
			X	Y	Z					Baseline vs. 5 s	Baseline vs. 30 min	5 s vs. 30 min
Classical	ORBinf	R	45	-24	–15	167	14.307	32.152	0.000	0.000	0.353	0.000
	Calcarine	L	-24	-60	-6	989	17.429	11.688	0.000	0.148	0.000	0.000
	MOG	R	39	-87	0	191	27.354	22.704	0.000	0.000	0.000	0.003
	MFG	L	6	57	12	1262	20.011	32.378	0.000	0.000	0.000	0.122
	IFGtriang	R	63	12	12	238	14.555	36.949	0.000	0.000	0.375	0.000
	AnG	L	-42	-54	33	485	16.106	30.137	0.000	0.000	0.000	0.099
	Precuneus	L	0	-69	36	420	22.444	23.043	0.000	0.000	0.000	0.824
	MFG	R	36	45	39	158	15.272	23.383	0.000	0.004	0.000	0.000
	PCG	L	-24	-54	60	676	18.831	18.269	0.000	0.871	0.000	0.000
Slow 5	MOG	R	45	-75	-6	102	14.176	19.987	0.000	0.002	0.000	0.000
	MFG	L	-33	51	27	128	14.667	18.201	0.000	0.000	0.000	0.103
	Precuneus	L	0	-51	42	96	10.187	11.225	0.000	0.000	0.000	0.280
Slow 4	ORBinf	R	48	18	-18	259	14.269	40.362	0.000	0.000	0.108	0.000
	Calcarine	L	-15	-54	3	940	18.047	11.135	0.000	0.215	0.000	0.000
	MFG	L	-12	63	9	1053	21.368	37.574	0.000	0.000	0.000	0.078
	Precuneus	L	0	-66	36	488	21.142	21.134	0.000	0.000	0.000	0.126
	AnG	L	-51	-54	27	304	14.108	26.959	0.000	0.000	0.000	0.929
	PCG	L	-33	-27	51	731	17.263	19.105	0.000	0.786	0.000	0.000
Slow 3	Calcarine	L	0	-69	18	1144	19.932	12.598	0.000	0.176	0.000	0.000
	MFG	R	42	48	33	125	16.567	19.724	0.000	0.163	0.000	0.000
	SPG	L	-24	-54	60	124	19.087	17.073	0.000	0.665	0.000	0.000
Slow 2	SOG	L	-21	-90	27	52	12.117	10.138	0.000	0.965	0.000	0.000

*CTN, Classical trigeminal neuralgia; MNI, Montreal Neurological Institute; Baseline, the rs-fMRI was performed before stimulating the trigger zone; 5 s, the rs-fMRI was performed within 5 s after stimulating the trigger zone; 30 min, the rs-fMRI was performed in the 30th minute after stimulating the trigger zone; ORBinf, inferior frontal gyrus, orbital part; MOG, Middle occipital gyrus; MFG, Middle frontal gyrus; AnG, angular gyrus; PCG, postcentral gyrus; IFGtriang, inferior frontal gyrus, triangular part; SOG, Superior occipital gyrus; SPG, Superior parietal gyrus; R, right; L, left*

Except the left precuneus in slow 5 frequency band, the changed trend of the same brain regions in other frequency band were consistent with that in classical frequency band. In addition, a specific brain region was found in slow 3 (left superior parietal gyrus) and slow 2 (left superior occipital gyrus) frequency band respectively with the same changed trend, which were decreased at triggering-5 s and increased at triggering-30 min (Table 1 and S3, Figs. 1 and 2).

### dReHo changed in classical frequency band after triggering pain in CTN patients

The dReHo of the midbrain, left thalamus, and right putamen were decreased in triggering-5 s and increased at triggering-30 min, and gradually increased dReHo in the right anterior cingulate cortex (ACC) at triggering-5 s and triggering-30 min. There was no significant difference in the midbrain between triggering-5 s and triggering-30 min, and no significant difference in the left thalamus and right putamen between baseline and triggering-30 min. Also, there were significant differences among the other time points (Table 2 and S3, Figs. 1 and 2).

**Table 2:** The dynamic regional homogeneity difference in CTN patients after triggering pain

Brain region	Side	Peak MNI coordinates			Cluster size (voxels)	Peak intensity	F value	P value	Post hoc P value		
		X	Y	Z					Baseline vs. 5 s	Baseline vs. 30 min	5 s vs. 30 min
Midbrain		3	-30	-21	194	21.077	17.924	0.000	0.000	0.001	0.148
Putamen	R	21	-15	9	3396	46.034	56.782	0.000	0.000	0.103	0.000
ACC	R	6	42	18	200	14.702	11.111	0.000	0.032	0.000	0.001
Thalamus	L	-9	-12	6	157	17.481	19.008	0.000	0.000	0.936	0.000

*CTN, Classical trigeminal neuralgia; MNI, Montreal Neurological Institute; Baseline, the rs-fMRI was performed before stimulating the trigger zone; 5 s, the rs-fMRI was performed within 5 s after stimulating the trigger zone; 30 min, the rs-fMRI was performed in the 30th minute after stimulating the trigger zone; ACC, anterior cingulate cortex; R, right; L, left*

### Correlation between the demographics and indicators of altered brain region

**Fig. 3:**
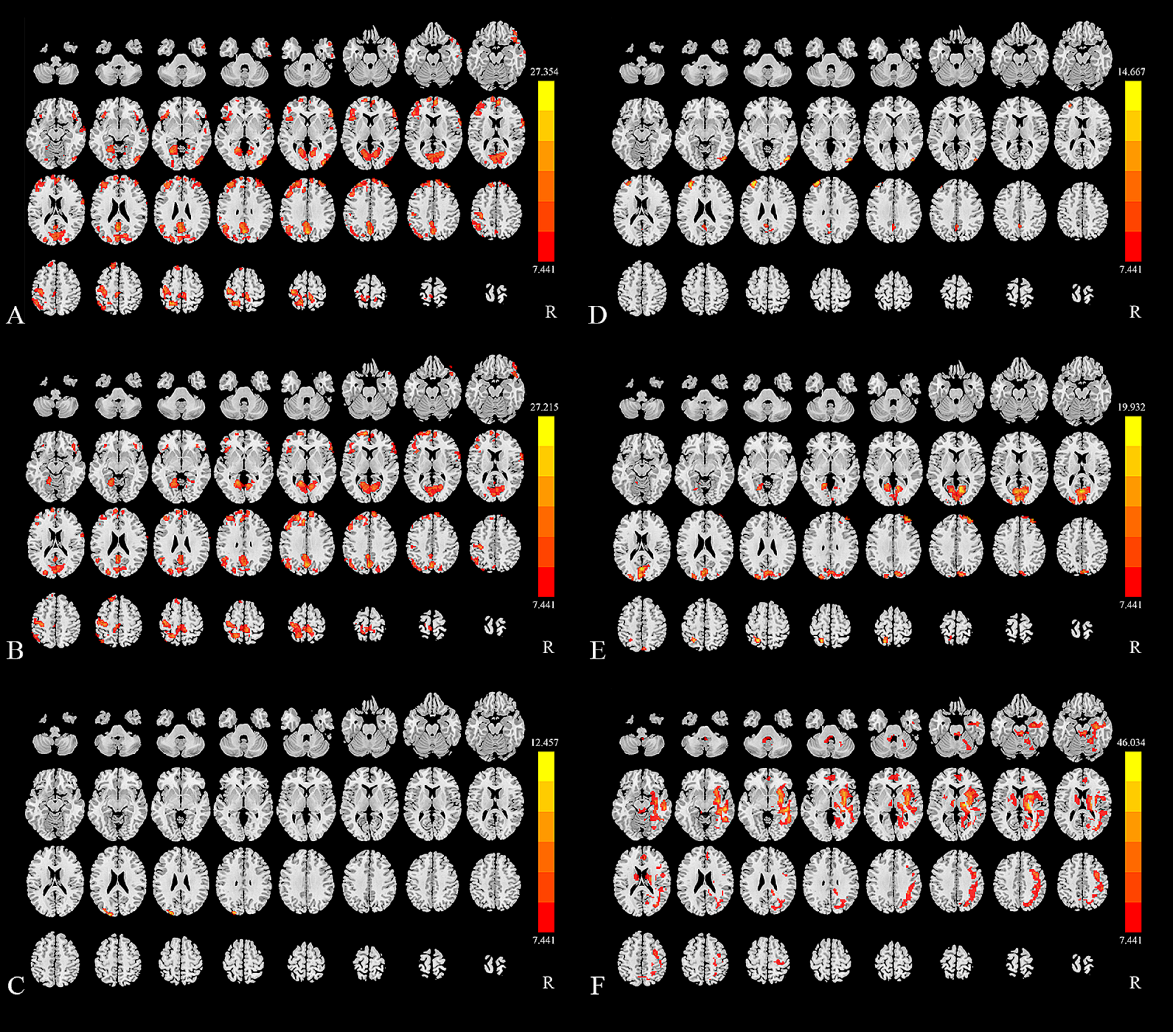
The correlation between the demographics and the indicators of altered brain region. **A** The sReHo value of the Precuneus.L at triggering-5 s in classical frequency band was negatively correlated with VAS; **B** The sReHo value of the Precuneus.L at baseline in slow 5 frequency band was negatively correlated with VAS; **C** The sReHo value of the MFG.L at baseline in slow 5 frequency band was negatively correlated with VAS; **D** The sReHo value of the MFG.L at triggering-5 s in slow 4 frequency band was negatively correlated with VAS; **E** The sReHo value of the AnG.L at triggering-5 s in slow 4 frequency band was negatively correlated with VAS; **F** The sReHo value of the Calcarine.L at triggering-5 s in slow 4 frequency band was negatively correlated with VAS. *sReHo, static regional homogeneity; VAS, Visual Analogue Scale; MFG, Middle frontal gyrus; AnG, angular gyrus; L, left*

Before the pain stimulated, the sReHo values of the Precuneus.L and MFG.L in slow 5 frequency band were negatively correlated with VAS respectively. The sReHo value of the Precuneus.L at triggering-5 s in classical frequency band was negatively correlated with VAS. The sReHo values of the MFG.L, AnG.L, and Calcarine.L at triggering-5 s in slow 4 frequency band were negatively correlated with VAS respectively (Fig. 3).

## Discussion

In this study, the key findings were as follows: (1) in the five frequency bands, the number of the altered brain regions in classical frequency band were most, followed by slow 4 frequency band, and the changing trend of the same brain region was consistent except the left precuneus in slow 5 frequency band; (2) there was a specific brain region was found in slow 3 (left superior parietal gyrus) and slow 2 (left superior occipital gyrus) frequency band respectively with the same change trend; (3) the changed brain regions of dReHo and sReHo were different, and complementary to each other; (4) there were four altered trends and dominated by decreased at triggering-5 s and increased at triggering-30 min were most.

### sReHo changed in classical frequency band after triggering pain

In classical frequency band, the sReHo changed in nine brain regions after a single pain stimulated and there were four changed trends, indicating that different brain regions have different functions in pain process.

The middle occipital gyrus and calcarine were the visual processing center in mammals [21] and participated in forming the visual networks [41]. The sReHo of right middle occipital gyrus and left calcarine were gradually increased at the two time points after triggering pain. This indicated that neuronal activity in the two regions tended to be time-synchronized after a single pain stimulate and there was no recovery trend. And there was no significant difference between the baseline and triggering-5 s in left calcarine, suggested that it changed slowly in a short time after pain stimulated. The pain experience was accompanied by some sensory input, such as vision [42]. The sReHo changed in the above two brain regions after pain stimulated indicated that visual cortex was involved in the pain process.

The default mode network (DMN) was the most stable resting-state network, and usually activated when there was no contact with the external environment [21, 43]. DMN activity decreased generally during engagement with tasks and stimuli (including painful stimuli) [44]. The sReHo values of the bilateral middle frontal gyrus and left precuneus were gradually decreased at triggering-5 s and triggering-30 min and no significant difference between the two time points in left middle frontal gyrus and precuneus. We speculated that the signal in the two brain regions recovered with time. Borsook et al. [45] found middle frontal gyrus was significantly activated before and after pain in a TN patient. Yuan et al. [21] showed the ReHo value of precuneus increased in TN patients compared with HCs. The results of the above researches were inconsistent with ours, which might because the basic conditions of subjects were different.

The angular gyrus and precuneus were participated in the constitution of DMN. The sReHo value of left angular gyrus decreased rapidly at triggering-5 s and then changed slowly, and the sReHo value of left precuneus was negatively correlated with VAS. The top-down regulation of pain by DMN may be coordinated by angular gyrus [46]. Therefore, we hypothesized the angular gyrus might involve in pain processing rapidly and then recovering slowly. The postcentral gyrus was the primary somatosensory cortex [47], which involved in the composition of the ascending injury pathway [48] and associated with the anticipation, intensity, discrimination, spatial, and temporal summation aspects of pain processing [43]. The changed trend of left postcentral gyrus was consistent with that of left angular gyrus and precuneus. This further suggested that local neuronal activity of left postcentral gyrus was slightly chaotic at triggering-5 s and then tended to synchronize at triggering-30 min.

The IFG belonged to prefrontal cortex and had an important role in emotional processing, cognitive processing, and pain management [49], which could divide into orbital, trigonometry, and operculum. The sReHo of right inferior frontal gyrus, orbital and triangular part increased at triggering-5 s and decreased at triggering-30 min. Although the sReHo values did not recover to the baseline at triggering-30 min, there was no significant difference between the two time points, which indicated that local neuronal activity of right inferior frontal gyrus, orbital part and triangular part had the recovery trends at triggering-30 min.

### sReHo changed in slow 2 to slow 5 frequency bands after triggering pain

The number of sReHo changed brain regions in other frequency bands were different. Compared with classical frequency band, there were one specificity brain region in slow 3 and slow 2 frequency bands respectively, and other altered brain regions were covered. The reason might that the frequency of classical frequency band completely included that of slow 4 and slow 5 frequency bands, partly included that of slow 3 frequency band, and different with slow 2 frequency band. Therefore, we hypothesized that local brain function has frequency-dependent characteristic in CTN patients after a single pain stimulate.

The superior parietal gyrus, calcarine, and middle occipital gyrus were located in occipital lobe and participated in forming the visual network. Previous studies have shown that the vision was involved in the integration of brain functions in chronic pain [50, 51]. The sReHo value of left superior parietal gyrus at triggering-5 s was similar to that at baseline (*P* = 0.965) and significantly increased at triggering-30 min in slow 2 frequency band. This was different at triggering-5 s and consistent at triggering-30 min with that of left calcarine and middle occipital gyrus in other frequency bands. This indicated a certain similarity in changed trend of brain regions with the same function in different frequency bands.

### dReHo changed after triggering-pain in classical frequency band

The dReHo changed in four brain regions after a single pain stimulate, and were completely different with that of sReHo, indicated that the dReHo could provide additional information and a new perspective for the central mechanisms of CTN patients. At present, dReHo research had been applied in TN [33, 34], depression [52], epilepsy [53] etc. One study found the dReHo decreased in left middle occipital gyrus, parietal lobules, and precentral gyrus and increased in thalamus in TN patients [33], yet, that study only conducted a single time point of comparative study.

The ACC participated in pain processing, cognition, and emotion [54], which was a part of pain matrix, and the signal of ACC increased during pain stimulation and decreased during analgesia [46, 50]. The dReHo value of right ACC increased gradually, indicated that ACC had a certain role in the pain process of CTN patients and the time variability increased gradually. Borsook et al. [45] found that the ACC was activated during both induced and spontaneous pain in TN patients.

The dReHo value of midbrain, left thalamus, and right putamen decreased at triggering-5 s and increased at triggering-30 min, and dReHo values of left thalamus and right putamen at triggering-30 min were similar to that at baseline. The midbrain changed slowly at triggering-5 s and triggering-30 min after a single pain stimulate, and there was no significant statistical difference between the two. The midbrain was a major site of nociceptive input, processing, and regulation [55], receiving signal from multiple regions, including thalamus and ACC [54]. The putamen and caudate were the main sites of basal ganglia cortical input, and the putamen involved in pain processing [55] and often activated during pain [21]. Moisset et al. [23] found that the pain stimulation could cause the activation of brain regions such as the thalamus, ACC, putamen, and midbrain by studying pain task state in CTN patients.

Previous studies found that the thalamus, midbrain, ACC, and putamen involved in the composition of the pain regulation system [25, 50, 56]. The brain regions which the dReHo changed were different with that of sReHo in multi-frequency bands. Therefore, we speculated the local function activity of changed brain regions in CTN patients had temporal characteristic.

## Conclusion

The duration of the brain function in altered brain regions were more than 30 min, although transient pain was one characteristic of CTN patients. Among the six frequency bands, the classical frequency band could be best explore the local brain function changing. The changed brain region had time-frequency characteristic in CTN patients after a single pain stimulate, and the brain regions of the time and frequency complemented to each other. The above findings could provid a certain theoretical basis for exploring the pathophysiology of CTN.

### Limitations

In this study, we performed scans 30 min after a single pain stimulate to explore whether the brain regions which the sReHo and dReHo values changed had returned to baseline. Apparently not, this required scanning at extended intervals to determine how long it would take for the consistency of the local brain tissue to return after triggering-pain. Depending on whether the pain accompanied by persistent pain or not, CTN were divided into two sub-types, and there might be certain differences in brain function between the two, which requires us to conduct further typing research. The third was that we did not do a subgroup study based on the symptoms and severity of CTN, in the future, we will expand the sample size and conduct the subgroup study. The last was that we only study the CTN patients who were pain after stimulating, in the future, we should analyzed the patients who did have pain after triggering.

**Highlights**.

First, our study was conducted by simulating or reproducing a single pain stimulate in CTN patients, and we obtained three rs-fMRI scans (baseline, triggering-5s, and triggering-30 min), which could reflect the changed trends of brain function well.

Second, we used multi-frequency bands of sReHo and dReHo to investigate time-frequency characteristics.

### Electronic supplementary material

Below is the link to the electronic supplementary material.

Supplementary Material 1

## Data Availability

The data and materials (including code) will be provided (Xiuhong Ge) to qualified researchers upon reasonable request.
