# Supplementary material for: Altered trends of local brain function in classical trigeminal neuralgia patients after a single trigger pain
Source: BMC Med Imaging. 2024 Mar 18;24:66. doi: 10.1186/s12880-024-01239-y (PMC10949736; doi:10.1186/s12880-024-01239-y)
Supplement: Supplementary file 1 — Supplementary Material 1 [file 12880_2024_1239_MOESM1_ESM.doc]

**Table S1**. The dynamic regional homogeneity difference in CTN patients after stimulated the pain (step size of 1 TRs (2 s)).

| Brain region | Side | Peak MNI coordinates | | | Cluster size  (voxels) | Peak intensity | *F* value | *P* value | *Post hoc* *P* value | | |
| --- | --- | --- | --- | --- | --- | --- | --- | --- | --- | --- | --- |
|  |  | X | Y | Z |  |  |  |  | Baseline vs 5 s | Baseline vs 30 min | 5 s vs 30 min |
| Midbrain |  | 3 | -30 | -21 | 195 | 20.646 | 17.319 | 0.000 | 0.000 | 0.000 | 0.149 |
| Putamen | R | 21 | -15 | 9 | 3367 | 46.564 | 56.413 | 0.000 | 0.000 | 0.105 | 0.000 |
| ACC | R | 6 | 42 | 18 | 196 | 14.116 | 11.040 | 0.000 | 0.047 | 0.000 | 0.000 |
| Thalamus | L | -9 | -12 | 6 | 150 | 17.159 | 18.497 | 0.000 | 0.000 | 0.968 | 0.000 |

*ACC, anterior cingulate cortex.*

**Table S2**. Demographics and behavioral results of CTN

|  | | CTN |
| --- | --- | --- |
| Men/Women | | 11 37 |
| Age (year) | | 55.65 ± 11.41 |
| Lateral | | 32R/16L |
| Duration (year) | | 3.0 (1.225–6.375) |
| Average duration of attack (min) | < 1 | 33 |
|  | 1 ~ 2 | 4 |
|  | > 2 | 11 |
| Pain location | V2.3 | 26 |
|  | V3 | 11 |
|  | V2 | 8 |
|  | V1.2 | 2 |
|  | V1.2.3 | 1 |
| Pain intensity (VAS) | | 7.85 ± 2.00 |

*CTN, Classical trigeminal neuralgia; VAS, Visual Analogue Scale.*

**Table S3**. The sReHo values in each frequency band and dReHo values in CTN patients after triggering the pain.

| Bands | Brain region | Side | sReHo value | | |
| --- | --- | --- | --- | --- | --- |
|  |  |  | Baseline | 5s | 30min |
| Classical | IFG-orb | R | 0.914 ± 0.088 | 0.981 ± 0.119 | 0.904 ± 0.105 |
|  | Calcarine | L | 1.220 ± 0.239 | 1.260 ± 0.266 | 1.404 ± 0.306 |
|  | MOG | R | 0.968 ± 0.101 | 1.032 ± 0.113 | 1.087 ± 0.153 |
|  | MFG | L | 1.292 ± 0.129 | 1.193 ± 0.116 | 1.169 ± 0.130 |
|  | IFG-Tri | R | 0.870 ± 0.044 | 0.944 ± 0.071 | 0.879 ± 0.063 |
|  | Angular | L | 1.518 ± 0.144 | 1.408 ± 0.142 | 1.436 ± 0.151 |
|  | Precuneus | L | 2.090 ± 0.262 | 1.899 ± 0.252 | 1.905 ± 0.243 |
|  | MFG | R | 1.123 ± 0.150 | 1.078 ± 0.140 | 1.026 ± 0.143 |
|  | Postcentral | L | 1.023 ± 0.155 | 1.020 ± 0.172 | 1.132 ± 0.194 |
| Slow-5 | MOG | R | 0.932 ± 0.136 | 1.006 ± 0.146 | 1.102 ± 0.194 |
|  | MFG | L | 1.354 ± 0.230 | 1.195 ± 0.193 | 1.152 ± 0.210 |
|  | Precuneus | L | 2.353 ± 0.397 | 2.131 ± 0.358 | 2.078 ± 0.346 |
| Slow-4 | ORBinf | R | 0.904 ± 0.062 | 0.973 ± 0.862 | 0.887 ± 0.073 |
|  | Calcarine | L | 1.234 ± 0.248 | 1.273 ± 0.280 | 1.436 ± 0.340 |
|  | MFG | L | 1.276 ± 0.123 | 1.178 ± 0.110 | 1.151 ± 0.128 |
|  | Precuneus | L | 1.908 ± 0.234 | 1.735 ± 0.223 | 1.779 ± 0.230 |
|  | Angular | L | 1.573 ± 0.192 | 1.436 ± 0.177 | 1.438 ± 0.195 |
|  | Postcentral | L | 1.024 ± 0.151 | 1.020 ± 0.159 | 1.137 ± 0.200 |
| Slow-3 | Calcarine | L | 1.184 ± 0.147 | 1.206 ± 0.178 | 1.312 ± 0.204 |
|  | MFG | R | 1.021 ± 0.085 | 1.006 ± 0.096 | 0.954 ± 0.104 |
|  | SPG | L | 1.080 ± 0.962 | 1.075 ± 0.120 | 1.156 ± 0.141 |
| Slow-2 | SOG | L | 1.029 ± 0.126 | 1.029 ± 0.109 | 1.133 ± 0.157 |
| dReHo | Midbrain |  | 0.277 ± 0.092 | 0.237 ± 0.069 | 0.246 ± 0.075 |
|  | Putamen | R | 0.186 ± 0.040 | 0.163 ± 0.036 | 0.182 ± 0.046 |
|  | ACC | R | 0.152 ± 0.043 | 0.161 ± 0.050 | 0.183 ± 0.068 |
|  | Thalamus | L | 0.151 ± 0.050 | 0.133 ± 0.037 | 0.152 ± 0.055 |

*CTN, Classical trigeminal neuralgia; sReHo, static regional homogeneity; Baseline, the rs-fMRI was performed before stimulating the trigger zone; 5 s, the rs-fMRI was performed within 5 s after stimulating the trigger zone; 30 min, the rs-fMRI was performed in the 30th minute after stimulating the trigger zone; ORBinf, inferior frontal gyrus, orbital part; MOG, Middle occipital gyrus; MFG, Middle frontal gyrus; AnG, angular gyrus; PCG, postcentral gyrus; IFGtriang, inferior frontal gyrus, triangular part; SOG, Superior occipital gyrus; SPG, Superior parietal gyrus; R, right; L, left; dReHo, dynamic regional homogeneity; ACC, anterior cingulate cortex.*


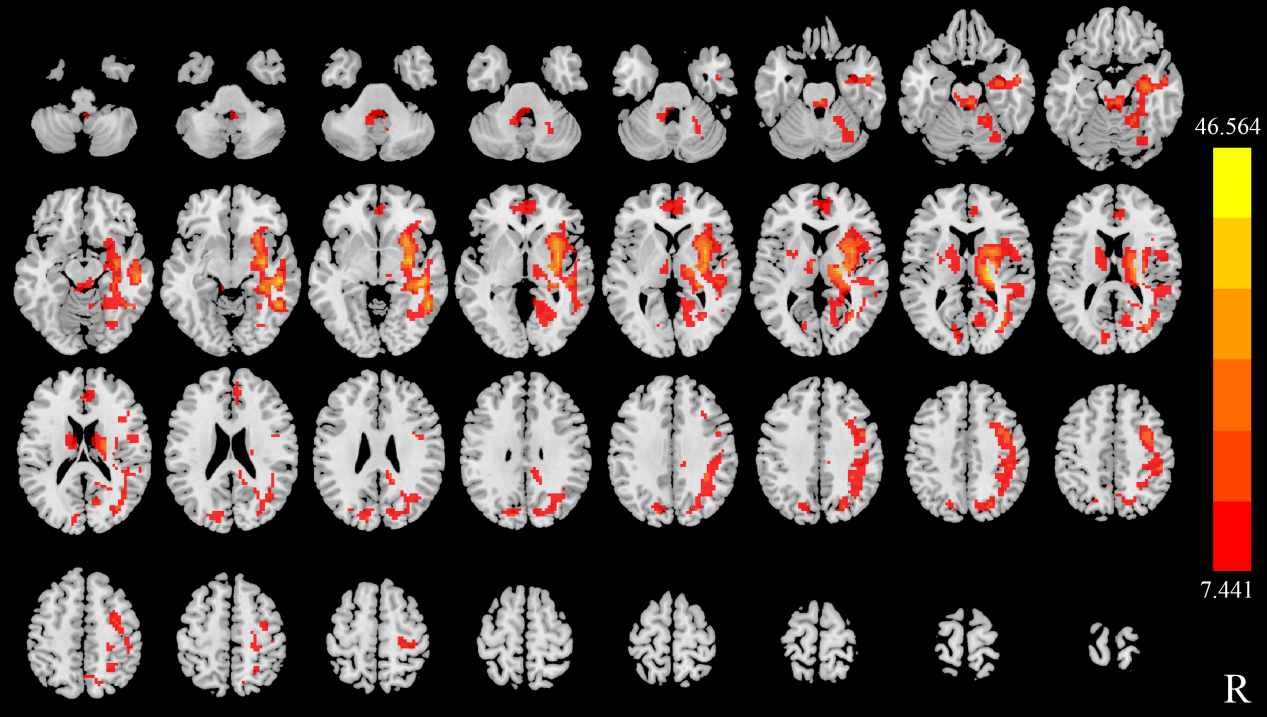
**Figure S1.** Significant differences in dReHo among different times after triggering the pain in patients with CTN. *dReHo, Dynamic regional homogeneity; CTN, classical trigeminal neuralgia.*


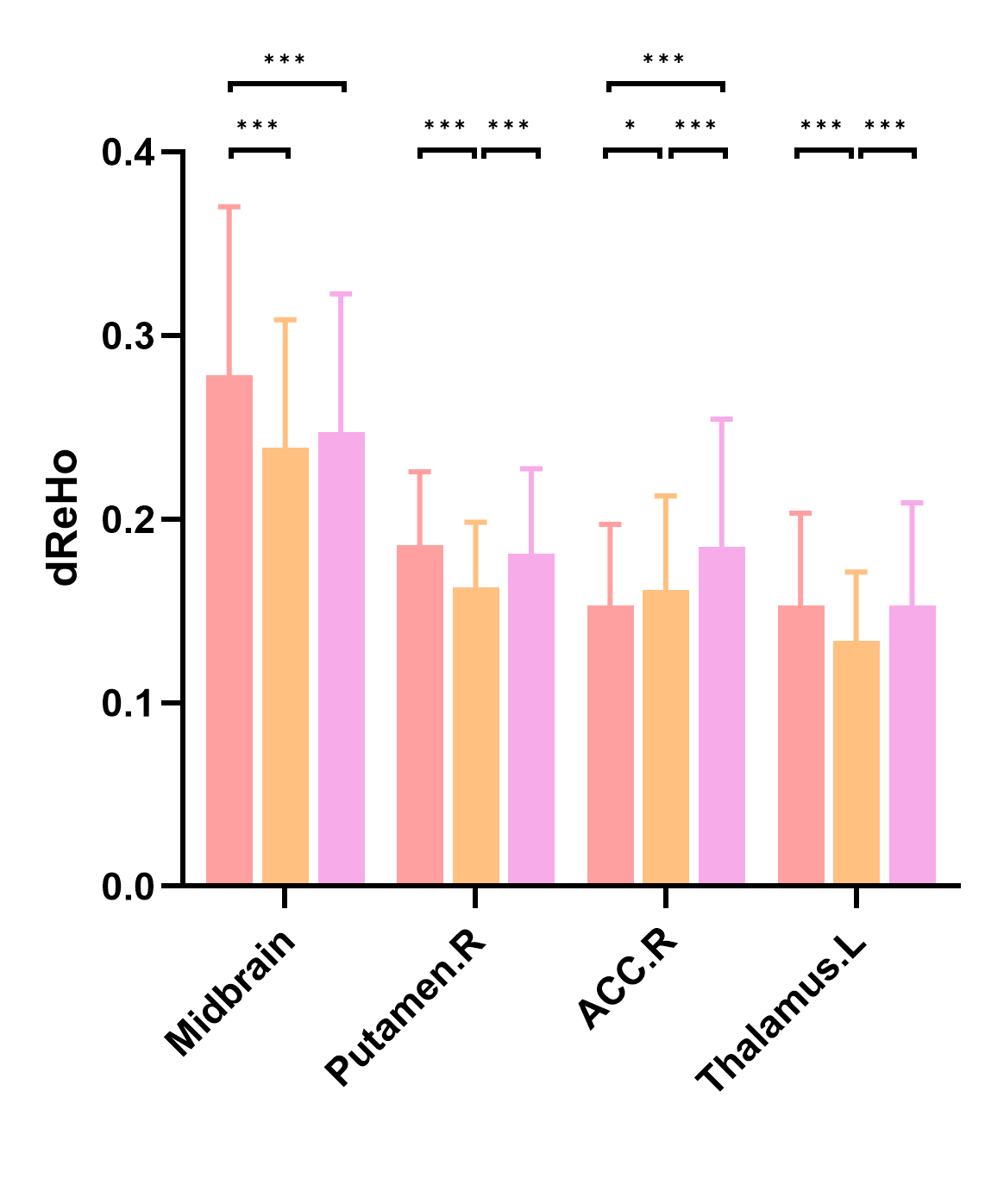


**Figure S2**. *Post hoc* comparisons of analysis of variance. *The connection between two bars represents significant between-time differences of dReHo (*represents significant level P < 0.05, **denotes significant level P < 0.01, and *** indicates significant level P < 0.001, Bonferroni correction). dReHo, Dynamic regional homogeneity; baseline, the rs-fMRI was performed before stimulating the trigger zone; triggering-5 s, the rs-fMRI was performed within 5 s after stimulating the trigger zone; triggering-30 min, the rs-fMRI was performed in the 30th minute after stimulating the trigger zone; Putamen.R, right putamen; ACC.R, right anterior cingulate cortex; Thalamus.L, left thalamus.*


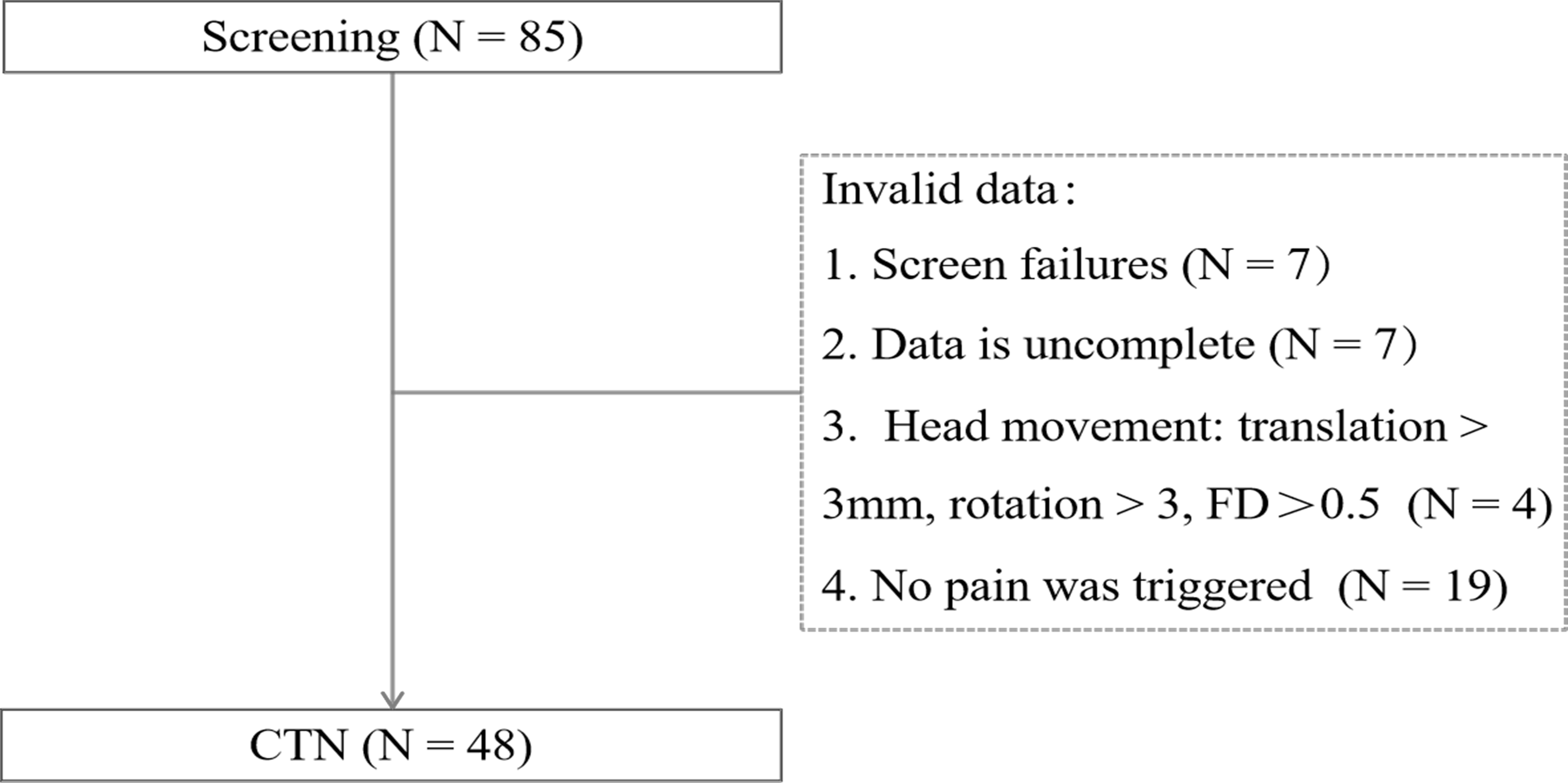


**Figure S3**. Participant selection. *FD, Framewise displacement; STN, secondary trigeminal neuralgia.*
